# Effects of olanzapine on resting heart rate in Japanese patients with schizophrenia

**DOI:** 10.1371/journal.pone.0199922

**Published:** 2018-07-17

**Authors:** Misuzu Tajiri, Yutaro Suzuki, Takuro Sugai, Nobuto Tsuneyama, Toshiyuki Someya

**Affiliations:** Department of Psychiatry, Niigata University Graduate School of Medical and Dental Sciences, Niigata, Japan; Chiba Daigaku, JAPAN

## Abstract

It has long been known that antipsychotic drugs (ATP) causes tachycardia, however details such as the differences between ATP are not well known. In recent years, the relationship between the rise in resting heart rate (RHR) and the increased risk of death in the general population has been garnering attention. In this study, we examined the difference in action on RHR between olanzapine (OLZ) and aripiprazole (ARP). The changes in the RHR on switching from OLZ to ARP and on increasing from the starting OLZ dose to the final one were evaluated in 19 outpatients (Study 1) and in 29 outpatients with schizophrenia (Study 2), respectively. To analyze the RHR, electrocardiographic measurements were obtained. At the same day, the Brief Psychiatric Rating Scale (BPRS) was evaluated, and fasting blood samples were drawn after an overnight fast of at least 8 h to examine electrolytes. Both Study 1 and 2 were conducted with the approval of the Gene Ethics Committee of Niigata University Graduate School of Medical and Dental Sciences, and the patients were treated at the outpatient psychiatric clinic at Niigata University Medical and Dental Hospital. All patients had been diagnosed with schizophrenia based on the DSM-IV-TR. In the Study 1, OLZ of 14.6 ± 9.2mg (mean ± standard deviation) was switched to ARP of 20.8 ± 8.1mg. Significant decreases were observed in the mean RHR after the switch to ARP (73.7 ± 9.7 vs 65.8 ± 10.9 beats/min, p = 0.008). In the Study 2, the starting OLZ dose was 7.2 ± 3.2mg and the increasing OLZ dose was 18.3 ± 7.4mg. Significant increases were observed in the mean RHR after increasing OLZ (69.7 ± 14.0 vs 75.6 ± 14.3 beats/min, p = 0.004). In this study, it was shown that OLZ has a stronger RHR enhancing effect compared to ARP and its effects are dose-dependent. If the increase in RHR increases the mortality rate of patients with schizophrenia, it may be necessary to further investigate the differences between ATP in terms of the effect on RHR of second-generation antipsychotics with a strong anticholinergic action or phenothiazine antipsychotics.

## Introduction

The average lifespan of patients with schizophrenia is short, a fact believed to be attributed to cardiovascular diseases partly due to the side effects of antipsychotic drugs (ATP) [1]. As a side effect of ATP for the circulatory system, much attention has been paid to the prolongation of electrocardiogram QT. However, while it has long been known that ATP causes tachycardia by blocking the muscarinic receptors present in the sinoatrial node or adrenaline α1 receptors, and details such as the differences between drugs are not well known. In recent years, the relationship between the rise in resting heart rate (RHR) and the increased risk of death in the general population has been garnering attention [2]. The median RHR in large-scale studies involving the general population was reported to be 68 in females and 66 in men [3]. It has also been reported that the risk of sudden cardiac death rises as RHR increases, along with the fact that while the occurrence rate of sudden cardiac deaths among the group with a RHR of 65 beats per minute or less is 1.1, the relative risk rises 5-fold or more among the group with a RHR of 80 beats/minute or more [4]. It was also reported that the high RHR was related to sudden cardiac death even in men without ischemic heart disease [5]. In addition, recent study showed that high RHR affects SCD even after adjusting the effects of left ventricular systolic dysfunction and drugs causing tachycardia such as β2-blocker and digoxin [6]. Of course, digoxin was also risk of independent SCD, but this study has not considered any other drugs such as ATP [6]. If ATP increase RHR, then this can be significantly problematic.

Although both olanzapine (OLZ) and aripiprazole (ARP) are widely used in the treatment of schizophrenia, the blocking actions thereof for adrenaline α_1_ receptors and muscarinic receptors differ. In particular, OLZ has stronger muscarinic receptor blocking action compared to ARP. Therefore, in this study, we examined the difference in action on RHR between OLZ and ARP, and investigated whether the effects of OLZ on RHR are dose dependent.

## Materials and methods

### Study 1

Study 1 was conducted in 19 outpatients between 18 and 60 years of age who had received a stable dose of OLZ monotherapy for ≥3 months for the treatment of schizophrenia based on the Diagnostic and Statistical Manual of Mental Disorders 4th edition text revision (DSM-IV-TR). Patients who did not obtain further improvement of symptoms by OLZ treatment were replaced with aripiprazole at the discretion of each clinical physicians. Each patient’s condition at entry had to be stable for inclusion in the study (i.e. no significant improvement or worsening of symptoms within the past 3 months). Patients did not have internal medical disorders that could affect heart rate, such as arrhythmia, heart disorder, endocrine disorder etc. Patients did not take any medications for medical disease. In addition, the patients did not take psychotropic medications such as antiparkinsonian and benzodiazepine agents. There was no change in the exercise volume, amount of smoking, daily meal during this study period.

At baseline, the Brief Psychiatric Rating Scale (BPRS) was evaluated, and fasting blood samples were drawn after an overnight fast of at least 8 h to examine electrolytes. The serum analyses were performed by standard methods (SRL, Inc., Tokyo, Japan). Electrocardiographic measurements were also obtained between 9:00 and 10:00 a.m. the same day. Following the baseline assessments, OLZ was switched to ARP at a starting dose of 6 mg/day while OLZ was tapered over 6 weeks. Clinical physicians of each patients decided the ARP treatment dose according to patient's condition for further improvement of clinical symptoms, respectively.

When the clinician confirmed no significant changing of symptoms with a stable dose of ARP for ≥3 weeks (endpoint), the same evaluations were conducted. All 19 patients who had been treated with OLZ were successfully switched from OLZ to ARP, and the duration of ARP treatment (from baseline to endpoint) was 12.5 ± 10.5 weeks (mean ± standard deviation [SD]).

### Study2

Study 2 was conducted in 29 outpatients who had been diagnosed with schizophrenia based on the DSM-IV-TR. All of the participants had been on a stable dose of OLZ monotherapy (starting dose) for ≥2 months. In Study2, patients who were not able to improve clinical symptoms any further by treatment with the same dose of OLZ were selected as subjects at the discretion of each clinical physicians. Patients did not have internal medical disorders that could affect heart rate, such as arrhythmia, heart disorder, endocrine disorder, etc. Patients did not take any medications for medical disease. In addition, the patients did not take psychotropic medications such as antiparkinsonian and benzodiazepine agents. There was no change in the exercise volume, amount of smoking, daily meal during this study period.

At baseline, the BPRS was evaluated, and fasting blood samples were drawn after an overnight fast of at least 8 h to examine electrolytes. Electrocardiographic measurements were also obtained between 9:00 and 10:00 a.m. the same day. Following the baseline assessments, the dose of OLZ was increased for further improvement of symptom. Clinical physicians of each patients decided the treatment dose according to patient's condition, respectively. The OLZ dose was adjusted differently for each patient based on clinical judgments. When the clinician confirmed no significant worsening of symptoms with a stable dose of OLZ (increased dose) for ≥3 weeks (endpoint), the same evaluations were conducted. All 29 patients who had been treated with OLZ were successfully switched from the starting dose to the increased dose.

A paired t-test was performed to evaluate the changes in the clinical and biochemical parameters on switching from OLZ to ARP (Study1) or on increasing from the starting OLZ dose to the final one (Study2). The SPSS software program, ver. 21.0 (IBM Japan, Ltd., Tokyo, Japan), was used for statistical analyses.

Both Study 1 and 2 were conducted with the approval of the Gene Ethics Committee of Niigata University Graduate School of Medical and Dental Sciences, and the patients were treated at the outpatient psychiatric clinic at Niigata University Medical and Dental Hospital. All patients were provided with thorough explanations of the experimental procedure prior to obtaining their written consent.

## Results

### Study 1

The clinical characteristics of the patients are shown in Table 1. The changes in the clinical and biochemical parameters after switching from OLZ to ARP are shown in Table 2. Significant decreases were observed in the mean body weight and RHR. In addition, no correlation was found between body weight and RHR, and it was found that body weight did not affect RHR in multiple regression analysis.

**Table 1:** Sample description of study1.

Variables	
Mean ± SD age (years)	30.2 ± 11.7
Male/female (n)	7/12
Mean ± SD duration of illness (years)	8.6 ± 9.3
Smoker/non-smoker (n)	6/13
Inpatients/outpatients (n)	6/13

SD = standard deviation

**Table 2:** Changes in resting heart rate and clinical parameters in switching from olanzapine to aripiprazole treatment.

	Olanzapine treatment	Aripiprazole treatment	*P* value
Dose (mg)	14.6 ± 9.2	20.8 ± 8.1	
Chlorpromazine equivalent doses (mg)	552.6 ± 374.7	521.1 ± 202.3	n.s.
BPRS	27.7 ± 8.0	26.5 ± 7.2	n.s.
Body weight (kg)	62.5 ± 13.5	59.6 ± 13.3	0.025
Waist (cm)	80.7 ± 12.5	78.1 ± 14.4	n.s.
Electrolyte (mEq/L)			
Na	140.3 ± 1.7	140.1 ± 1.7	n.s.
K	4.1 ± 0.3	4.2 ± 0.3	n.s.
Cl	104.3 ± 1.8	103.7 ± 1.9	n.s.
Mg	2.3 ± 0.1	2.3 ± 0.2	n.s.
Resting heart rate (beats/minute)	73.7 ± 9.7	65.8 ± 10.9	0.008
Systolic blood pressure (mmHg)	112.5 ± 15.2	112.5 ± 12.9	n.s.
Diastolic blood pressure (mmHg)	71.7 ± 9.1	67.2 ± 20.1	n.s.

All values are expressed as mean ± SD.

Abbreviations: BPRS = Brief Psychiatric Rating Scale.

### Study 2

The clinical characteristics of the patients are shown in Table 3. The changes in the clinical and biochemical parameters after increasing from the starting OLZ dose to the final one are shown in Table 4. Significant decreases were observed in the BPRS score, and significant increases were observed in the BMI and RHR. We did not find any associations between baseline/ change in/ endpoint resting heart rate and olanzapine dose. It was also found that body weight and BPRS did not affect RHR in multiple regression analysis.

**Table 3:** Sample description of Study2.

Variables	
Mean ± SD age (years)	26.7 ± 9.5
Male/female (n)	14/15
Smoker/non-smoker (n)	8/21

SD = standard deviation

**Table 4:** Changes in resting heart rate and clinical parameters in switching from starting-dose to increased-dose olanzapine treatment.

	Starting-dose treatment	Increased-dose treatment	*P* value
Dose (mg)	7.2 ± 3.2	18.3 ± 7.4	*P* < 0.001
BPRS	29.1 ± 7.3	23.4 ± 5.5	0.004
Body weight (kg)	59.6 ± 11.6	64.5 ± 12.8	0.001
Waist (cm)	79.1 ± 8.5	83.2 ± 14.2	n.s.
Electrolyte (mEq/L)			
Na	140.0 ± 1.7	140.5 ± 1.8	n.s.
K	4.2 ± 0.3	4.2 ± 0.2	n.s.
Cl	102.9 ± 2.8	104.3 ± 1.9	n.s.
Mg	2.3 ± 0.1	2.2 ± 0.2	n.s.
Resting heart rate (beats/minute)	69.7 ± 14.0	75.6 ± 14.3	0.004
Systolic blood pressure (mmHg)	115.5 ± 13.9	119.7 ± 20.6	n.s.
Diastolic blood pressure (mmHg)	72.6 ± 10.8	79.5 ± 15.1	n.s.

All values are expressed as mean ± SD.

Abbreviations: BPRS = Brief Psychiatric Rating Scale.

## Discussion

In this study, it was shown that OLZ has a stronger RHR enhancing effect compared to ARP and its effects are dose-dependent.

Although side effects of increasing in resting heart rate by ATP drug have been reported so far, details were unknown. To our knowledge, this study is the first of its kind showing the differences between ATP drugs in terms of their effects on enhancing RHR. The Ki values related to the receptor binding ability of OLZ and ARP were 19 and 57 nM, respectively, for adrenalin α_1_ receptors, indicating no significant difference; however, for muscarinic M_1_ receptors, the values were 1.9 and 10000 nM or more, respectively, indicating a significant difference [7–8]. From the above, the RHR enhancing effect of OLZ is thought to be caused by the stronger blocking effect of muscarinic receptors existing in the sinoatrial node of the heart compared to ARP. The α_1_ receptor blocking effect causes a decrease in blood pressure and the reactivity thereof may also result in a rise in heart rate. However, the potential for this is believed to be low. As evidence, in this study, before and after replacing ARP with OLZ (Table 2) as well as before and after changing the OLZ dose from low to high (Table 4), the systolic/diastolic blood pressure did not change.

There were studies that heart rate has increased as people gain weight [9–10]. Conversely, recent study showed that the presence or absence of obesity did not affect RHR in patients with schizophrenia [11]. In any case, the change in body weight did not affect RHR in this study.

Until now, studies using power spectrum analysis of heart rate variation as an indicator of autonomic nervous function have shown that ATP lower the function of both the sympathetic and parasympathetic nerves of schizophrenia patients [12]. According to these studies, heart rate and electrocardiogram RR fluctuations, along with heart rate and high-frequency content, which is considered to be an indicator of parasympathetic nervous activity, are negatively correlated [13]. These effects are reported to be particularly strong in ATP with strong anticholinergic action [14] as well as ATP at high doses [15]. In particular, clozapine with strong anticholinergic action has been shown to reduce parasympathetic nervous activity and increase RHR [16]. The results of this study, that the administration of OLZ, with strong anticholinergic action as clozapine, increased more RHR than ARP administration, support the result that until now autonomic nervous activity has been decreased by the administration of ATP.

However, limitation of this study is small sample size. Further study using a larger sample size should be needed.

Up to now, no attention has been paid to the effects of raising the heart rate using ATP. If the increase in RHR increases the mortality rate of patients with schizophrenia, it may be necessary to further investigate the differences between ATP in terms of the effect on RHR of second-generation antipsychotics with a strong anticholinergic action or phenothiazine antipsychotics.

## References

[1] Hennekens CH, Hennekens AR, Hollar D, Casey DE. Schizophrenia and increased risks of cardiovascular disease. Am Heart J. 2005; 150: 1115–21. 10.1016/j.ahj.2005.02.007 16338246

[2] Dyer AR, Persky V, Stamler J, Paul O, Shekelle RB, Berkson DM, et al Heart rate as a prognostic factor for coronary heart disease and mortality: findings in three Chicago epidemiologic studies. Am J Epidemiol. 1980; 112(6): 736–749. 7457467 10.1093/oxfordjournals.aje.a113046

[3] Mason JW, Ramseth DJ, Chanter DO, Moon TE, Goodman DB, Mendzelevski B. Electrocardiographic reference ranges derived from 79,743 ambulatory subjects. J Electrocardiol. 2007; 40 (3): 228–34. 10.1016/j.jelectrocard.2006.09.003 17276451

[4] Kannel WB, Kannel C, Paffenbarger RS Jr., Cupples LA. Heart rate and cardiovascular mortality. The Framingham Study. Am Heart J. 1987; 113 (6): 1489–94. 3591616 10.1016/0002-8703(87)90666-1

[5] Shaper AG, Wannamethee G, Macfarlane PW, Walker M. Heart rate, ischemic heart disease, and sudden cardiac death in middle-aged British men. British Heart Journal. 1993; 70: 49–55. 8037998 10.1136/hrt.70.1.49 PMC1025228

[6] Teodorescu C, Reinier K, Uy-Evanado A, Gunson K, Jui J, Chugh SS. Resting heart rate and risk of sudden cardiac death in the general population: influence of left ventricular systolic dysfunction and heart rate-modulating drugs. Heart Rhythm. 2013; 10 (8): 1153–8. 10.1016/j.hrthm.2013.05.009 23680897 PMC3765077

[7] ABILIFY [package insert]. Princeton NJ. Bristol-Myers Squibb and Rockville, MD. Otsuka America Pharmaceutical, 2005.

[8] Bymaster FP, Calligaro DO, Falcone JF, Marsh RD, Moore NA, Tye NC, et al Radioreceptor binding profile of the atypical antipsychotic olanzapine. Neuropsychopharmacology. 1996; 14 (2): 87–96. 10.1016/0893-133X(94)00129-N 8822531

[9] Frank S, Colliver JA, Frank A. The electrocardiogram in obesity: statistical analysis of 1,029 patients. J Am Coll Cardiol. 1986; 7(2): 295–9. 3944347 10.1016/s0735-1097(86)80494-6

[10] Rossi RC, Vanderlei LC, Gonçalves AC, Vanderlei FM, Bernardo AF, Yamada KM, et al Impact of obesity on autonomic modulation, heart rate and blood pressure in obese young people. Auton Neurosci. 2015; 193: 138–41. 10.1016/j.autneu.2015.07.424 26260435

[11] Sarlon J, Habich O, Schneider B. Elevated Rest Heart Rate in Psychiatric Patients and Different Effects of Psychotropic Medication. Pharmacopsychiatry. 2016; 49(1): 18–22. 10.1055/s-0035-1565204 26629642

[12] Alvares GA, Quintana DS, Hickie IB, Guastella AJ. Autonomic nervous system dysfunction in psychiatric disorders and the impact of psychotropic medications: a systematic review and meta-analysis. J Psychiatry Neurosci. 2016; 41(2): 89–104. 10.1503/jpn.140217 26447819 PMC4764485

[13] Billman GE, Huikuri HV, Sacha J, Trimmel K. An introduction to heart rate variability: methodological considerations and clinical applications. Front Physiol. 2015; 6: 55 10.3389/fphys.2015.00055 25762937 PMC4340167

[14] Huang WL, Chang LR, Kuo TB, Lin YH, Chen YZ, Yang CC. Impact of antipsychotics and anticholinergics on autonomic modulation in patients with schizophrenia. J Clin Psychopharmacol. 2013; 33 (2): 170–7. 10.1097/JCP.0b013e3182839052 23422372

[15] Iwamoto Y, Kawanishi C, Kishida I, Furuno T, Fujibayashi M, Ishii C, et al Dose-dependent effect of antipsychotic drugs on autonomic nervous system activity in schizophrenia. BMC Psychiatry. 2012; 14; 12: 199 10.1186/1471-244X-12-199 23151241 PMC3534356

[16] Agelink MW, Majewski T, Wurthmann C, Lukas K, Ullrich H, Linka T, et al Effects of newer atypical antipsychotics on autonomic neurocardiac function: a comparison between amisulpride, olanzapine, sertindole, and clozapine. J Clin Psychopharmacol. 2001; 21 (1): 8–13. 11199953 10.1097/00004714-200102000-00003

